# Plasma CCN2/connective tissue growth factor is associated with right ventricular dysfunction in patients with neuroendocrine tumors

**DOI:** 10.1186/1471-2407-10-6

**Published:** 2010-01-06

**Authors:** Deidi Strickland Bergestuen, Jørgen Gravning, Kristina Hermann Haugaa, Laura G Sahakyan, Svend Aakhus, Espen Thiis-Evensen, Erik Øie, Pål Aukrust, Håvard Attramadal, Thor Edvardsen

**Affiliations:** 1 Section of Gastroenterology, Department of Medicine, Oslo University Hospital, Rikshospitalet, Sognsvannsveien 20, 0027 Oslo, Norway; 2 Department of Cardiology, Oslo University Hospital, Rikshospitalet, Sognsvannsveien 20, 0027 Oslo, Norway; 3 Institute for Surgical Research, Oslo University Hospital, Rikshospitalet, Sognsvannsveien 20, 0027 Oslo, Norway; 4 Section of Immunology and Infectious Diseases, Department of Medicine, Oslo University Hospital Rikshospitalet, Sognsvannsveien 20, 0027 Oslo, Norway; 5 Research Institute for Internal Medicine, Oslo University Hospital, Rikshospitalet, Sognsvannsveien 20, 0027 Oslo, Norway; 6 Faculty of Medicine, University of Oslo, P.O. Box 1078 Blindern, 0316 Oslo, Norway

## Abstract

**Background:**

Carcinoid heart disease, a known complication of neuroendocrine tumors, is characterized by right heart fibrotic lesions. Carcinoid heart disease has traditionally been defined by the degree of valvular involvement. Right ventricular (RV) dysfunction due to mural involvement may also be a manifestation. Connective tissue growth factor (CCN2) is elevated in many fibrotic disorders. Its role in carcinoid heart disease is unknown. We sought to investigate the relationship between plasma CCN2 and valvular and mural involvement in carcinoid heart disease.

**Methods:**

Echocardiography was performed in 69 patients with neuroendocrine tumors. RV function was assessed using tissue Doppler analysis of myocardial systolic strain. Plasma CCN2 was analyzed using an enzyme-linked immunosorbent assay. Mann-Whitney U, Kruskal-Wallis, Chi-squared and Fisher's exact tests were used to compare groups where appropriate. Linear regression was used to evaluate correlation.

**Results:**

Mean strain was -21% ± 5. Thirty-three patients had reduced RV function (strain > -20%, mean -16% ± 3). Of these, 8 had no or minimal tricuspid and/or pulmonary regurgitation (TR/PR). Thirty-six patients had normal or mildly reduced RV function (strain ≤ -20%, mean -25% ± 3). There was a significant inverse correlation between RV function and plasma CCN2 levels (r = 0.47, p < 0.001). Patients with reduced RV function had higher plasma CCN2 levels than those with normal or mildly reduced RV function (p < 0.001). Plasma CCN2 ≥ 77 μg/L was an independent predictor of reduced RV function (odds ratio 15.36 [95% CI 4.15;56.86]) and had 88% sensitivity and 69% specificity for its detection (p < 0.001). Plasma CCN2 was elevated in patients with mild or greater TR/PR compared to those with no or minimal TR/PR (p = 0.008), with the highest levels seen in moderate to severe TR/PR (p = 0.03).

**Conclusions:**

Elevated plasma CCN2 levels are associated with RV dysfunction and valvular regurgitation in NET patients. CCN2 may play a role in neuroendocrine tumor-related cardiac fibrosis and may serve as a marker of its earliest stages.

## Background

Neuroendocrine tumors (NETs) are derived from the diffuse neuroendocrine cell system which is made up of cells that release hormones in response to signals from the nervous system. NETs most commonly arise from the gastrointestinal tract and the bronchopulmonary system. Carcinoid heart disease (CHD) is a known complication of these tumors, particularly of those arising from the small intestine, appendix and proximal colon (previously known as mid-gut carcinoids). CHD is characterized by plaque-like subendothelial lesions which are caused by proliferation of myofibroblasts and increased deposition of extracellular matrix [1,2]. These lesions result in retraction and fixation of the heart valves which in some cases may necessitate surgery.

Although CHD affects both mural and valvular endocardium, the condition has traditionally been defined by the degree of valvular involvement present most commonly in the form of tricuspid regurgitation. The criteria for CHD varies between studies with some including patients with at least mild or greater valvular involvement [2,3], while others limit the criteria to moderate or severe valvular pathology [4]. However, impaired right ventricular (RV) function due to fibrosis of the mural wall could potentially be an earlier manifestation of CHD.

Connective tissue growth factor (CCN2) is a 38 kDa cystein-rich secreted immediate early gene product and is a member of the CCN (Cyr61/CEF-10, CTGF/Fisp-12 and Nov) family of matricellular proteins. These proteins function as adaptor molecules linking the cell surface with extracellular matrix and act via integrins and proteoglycans [5,6]. CCN2 is upregulated in many fibrotic disease processes including hepatic and renal fibrosis, radiation enteritis and systemic sclerosis [7-11]. In addition, animal studies have shown increased expression of CCN2 in various forms of myocardial fibrosis including diabetic cardiomyopathy, pressure-overload cardiac hypertrophy and in ischemic heart failure [12-14]. Moreover, expression of CCN2 mRNA is significantly elevated in human heart tissue from patients with cardiac ischemia compared to normal controls [15]. It has been shown that plasma CCN2 is elevated in patients with small intestinal NETs compared to healthy controls, and that patients with small intestinal NETs associated with peritoneal fibrosis have increased tumor expression of CCN2 compared to patients without evidence of intraabdominal fibrosis [16]. However, at present, it is not known what role CCN2 may play in the development of CHD.

Assessment of myocardial strain is an emerging echocardiographic tool for evaluating RV function and has been validated in clinical studies [17-19]. It is not known whether there exists a relationship between CCN2 and RV function. Furthermore, to our knowledge, no published studies using assessment of strain to evaluate RV function in NET patients exist in the current literature.

We hypothesized that CCN2 could be involved in the pathogenesis of CHD, and in the current study, this hypothesis was examined by different experimental approaches. First, we measured plasma levels of CCN2 in a well-characterized population of patients with NETs and in sex- and age-matched healthy controls. Second, we examined the relationship between plasma levels of CCN2 and the degree of tricuspid or pulmonary valve involvement in the patient group. Finally, we assessed the relationship between plasma levels of CCN2 and RV function by means of RV lateral strain measurements in NET patients, with and without right-sided valvular pathology.

## Methods

### Selection of patients

Eighty-seven consecutive patients with histologically verified small intestinal (n = 84), appendiceal (n = 2) or proximal colonic (n = 1) NETs admitted to the gastroenterology ward between September 1, 2006 and September 1, 2007, for either first-time evaluation of a newly diagnosed NET or for follow-up, were screened for inclusion in this prospective study. Exclusion criteria included the presence of another fibrotic disease such as systemic sclerosis, age < 18 or the inability to give informed consent. We wanted to focus on the possible influence of active NETs, and accordingly, patients who had undergone radical surgery were not included in the study. Thus, only patients with residual tumor were included, resulting in a total number of 69 out of 87 screened patients. A 24-hour urine collection for 5-hydroxyindoleacetic acid (U-5HIAA) (normal range 0.8-3.8 μmol/mmol creatinine) was obtained. All of the patients routinely underwent CT examination of the abdomen as part of their admission (separate from the study). In order to assess tumor burden, reanalysis of the abdominal CT scans was performed to determine the number and size of liver metastases for each patient. The maximal diameter of each liver metastasis was determined and then the average maximal diameter of the total number of liver metastases for each patient was calculated. For comparison of plasma levels of CCN2, 18 age-and sex-matched healthy controls were also included. Demographic, clinical and tumor characteristics are given in table 1.

**Table 1:** Clinical and Tumor Characteristics

Characteristic*	Normal/mildly Reduced RV Function† (n = 36)	Reduced RV Function (n = 33)	P-value	No/minimal TR/PR‡ (n = 29)	Mild to Severe TR/PR (n = 40)	P-value
**Gender**			**0.06**			**0.22**
***Men***	13 (36%)	20 (61%)		11 (38%)	22 (55%)	
***Women***	23 (64%)	13 (39%)		18 (62%)	18 (45%)	
**Age§**	63 (36-81)	65 (33-83)	**0.87**	60 (36-75)	68 (33-83)	**<0.001**
**Smoking**	5 (15%)	9 (28%)	**0.23**	5 (17%)	9 (24%)	**0.56**
**Illness duration (yrs)**	3.4 (0.8-7.5)	5.1 (2.3-6.8)	**0.33**	2.3 (0.7-5.2)	5.3 (2.6-7.8)	**0.01**
**1° tumor size (mm)** ∥	20 (15-27)	20 (15-30)	**0.44**	20 (16-30)	19 (15-29)	**0.53**
**Carcinoid syndrome¶**	30 (83%)	29 (88%)	**0.74**	21 (72%)	38 (95%)	**0.01**
**Ki 67#**	2% (0-10)	2.5% (1-25)	**0.16**	2% (1-10)	2% (0-25)	**0.20**
U-5HIAA**	6 (2-19)	19 (4-29)	**0.08**	7 (2-19)	10 (3-30)	**0.21**
**CgA††**	14 (6-24)	26 (12-82)	**0.02**	16 (6-37)	19 (9-79)	**0.31**
**Liver metastases**	30 (83%)	30 (91%)	**0.48**	27 (93%)	33 (83%)	**0.29**
***Size (mm)*‡‡**	15 (7-19)	15 (13-25)	**0.18**	14 (9-19)	17 (11-24)	**0.22**
***Number***	6 (1-12)	11 (6-20)	**0.01**	6 (1-10)	11 (5-20)	**0.03**

*Results given as median (interquartile range) or n (%) unless otherwise specified. †Right ventricular function. ‡Tricuspid and/or pulmonary regurgitation. §Median (range). ∥ Maximal diameter of the primary tumor. ¶Defined as flushing and/or diarrhea. #Proliferation index given as median (range). **Urinary 5-hydroxyindoleacetic acid (normal 0.9-3.8 μmol/mmol creatinine). ††Chromogranin A (normal < 4 nmol/L). ‡‡Median maximal diameter.

### Blood sampling protocol

Peripheral venous blood was drawn from all patients and controls into pyrogen-free blood collection vials with EDTA as the anticoagulant, immediately immersed in ice and centrifuged within 1 hour at 2500 g for 20 minutes to obtain platelet-poor plasma. All samples were stored at -80°C and thawed only once.

### Biochemical measurements

Plasma levels of CCN2 were measured using a sandwich enzyme-linked immunosorbent assay (ELISA). Briefly, assay plates were coated with monoclonal anti-human CCN2 IgG_1 _raised against a peptide from the carboxyl-terminal domain of CCN2 (MAB660, R&D Systems, Abingdon, UK) and incubated at 4°C overnight (20 μg/ml in phosphate buffered saline [PBS]). After blocking nonspecific binding sites for 2 hours with blocking buffer containing 1% bovine serum albumin (BSA), 100 μL of plasma samples diluted in 100 μL assay buffer (containing 50 mM Tris pH 7.8, 0.1% BSA, Triton X-100 and heparin) were added in duplicates to the microwells. Standard curve was obtained from dilutions of recombinant human CCN2 in 50% calf serum, contract-purified by EMP Genetech (Ingolstadt, Germany). The plates were then incubated overnight at 4°C with gentle shaking, washed with PBS including 0.5 ml/L Tween 20, before biotinylated goat anti-human CCN2 IgG (BAF660, R&D Systems) (2 μg/ml) was added and incubated for 2 hours with shaking. Plates were washed, followed by incubation with streptavidin-HRP (DakoCytomation, Glostrup, Denmark) and finally, substrate reagent TMB (R&D Systems) was added. Color development was stopped after 20 minutes at 37°C with H_2_SO_4_. Absorbance was measured at 450 nm. The calibration curve was fitted through use of a 5-parameter logistic function (r^2 ^= 0.999). The intra-assay variation (CV) was 8.7% (125 μg/L). The lower limit of detection was 7.8 μg/L. After dilution, 5 of the samples still had CCN2 levels above the upper limit of detection (4000 μg/L) and accordingly, were assigned this value. All samples were analyzed simultaneously. Patient blood samples were additionally analyzed for chromogranin A (CgA), a known tumor marker for neuroendocrine tumors [20], using a radioimmunoassay provided by EuroDiagnostica (Malmö, Sweden) (normal <4 nmol/L).

### Echocardiographic Examination

**Figure 1:**
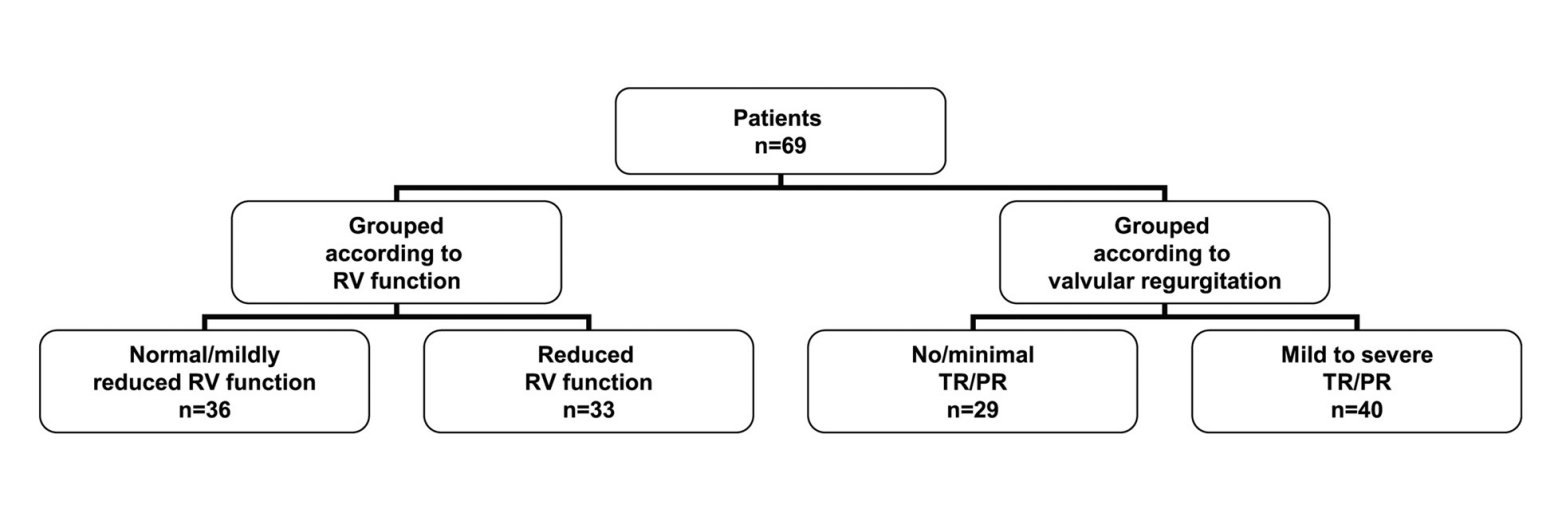
**Patient subgroups**. Flow chart showing patient subgroups based on either right ventricular (RV) function or the absence or presence of right-sided valvular regurgitation (TR/PR = tricuspid and/or pulmonary regurgitation).

Patients were evaluated with transthoracic 2-dimensional, M-mode and Doppler echocardiography using Vivid 7 (GE Healthcare, Horten, Norway) and analyzed with commercially available software (EchoPAC^®^, GE). Two cardiologists well experienced in echocardiography reviewed the echocardiographic registrations off-line on a dedicated work station (EchoPacPC, GE Vingmed). They were blinded to patients' symptoms, clinical status and biochemical data. The severity of valvular regurgitation was determined according to standard guidelines [21,22]. Valvular regurgitation was graded as mild, mild-to-moderate, moderate or severe [22]. CHD was defined as the presence of right-sided valvular regurgitation or stenosis (excluding both trivial or mild findings [23]) plus morphologic changes including thickening, shortening, retraction, hypomobility or incomplete coaptation of the affected valves [2,4]. In order to assess the relationship between CCN2 and impaired RV strain as a possible early manifestation of CHD, the patients were further divided into groups with either no or minimal valvular involvement or at least mild or greater valvular involvement of the right-sided valves (Figure 1).

### 2D-strain echocardiography

2-D grayscale images were acquired in the standard apical views at 67 ± 26 frames/s. Three cardiac cycles were recorded. Myocardial deformation measurements were performed using speckle tracking echocardiography [24-27]. RV longitudinal systolic strain was obtained from the apical four chamber view. Mean systolic strain from the three lateral segments of the RV was calculated. As there currently exists no universal criteria for defining normal and reduced RV function using RV mean lateral strain values, we used the median to determine our cut-off value of -20%. However, data from a recent study lend support to the rationality of this cut-off value. In this study, peak systolic strain in healthy individuals was -29% ± 6, in endurance athletes (RV dilatation but normal RV function) -27% ± 7, and in patients with impaired RV function (global or regional hypokinesia of the RV free wall) -17% ± 7 [28]. In another study, RV mean lateral strain in 32 control subjects was -32% ± 12 [29]. Consequently, in the present study, patients with mean lateral strain ≤ -20% were defined as having normal or mildly reduced RV function, while those with strain > -20% were defined as having reduced RV function (Figure 1).

### Immunohistochemistry

Biopsies of the primary tumor from five of the patients and of a liver metastasis from two of the patients were retrieved from pathology archives. All seven patients had small intestinal primary tumors and widespread liver metastases. Three of these patients had reduced RV function and five had at least mild or greater tricuspid and/or pulmonary regurgitation (TR/PR). Four of the patients fulfilled the criteria for CHD. Formalin-fixed sections of gut and liver NET tissue were stained using polyclonal rabbit anti-rat CCN2 [12] and anti-human CgA (Santa Cruz Biotechnology, CA) IgG and anti-human smooth muscle actin (DakoCytomation). The primary antibodies were followed by biotinylated anti-rabbit or anti-mouse IgG (Vector Laboratories, Burlingame, CA). The immunoreactivities were further amplified using avidin-biotin-peroxidase complexes (Vectastain Elite kit, Vector Laboratories). Diaminobenzidine was used as the chromogen in a commercial metal-enhanced system (Pierce Chemical, Rockford, IL). The sections were counterstained with hematoxylin. Omission of the primary antibody served as negative control.

### Ethics

The study complies with the Helsinki Declaration and was approved by both the Institutional Review Board and the Regional Ethics Committee for Research. All individuals gave their written informed consent to participate in the study.

### Statistical analyses

Data for continuous variables are presented as either median with the interquartile range or mean ± SD based on their distribution unless otherwise specified. Mann-Whitney U, Kruskal-Wallis, χ^2 ^and Fisher's exact tests were used to compare groups where appropriate. Because of extreme outliers, logarithmic transformation of plasma CCN2 values was performed before presentation of data in the figures. Log10 transformation values of plasma CCN2 were used in linear regression analysis. For all other comparisons of plasma CCN2 levels, non-transformed data were employed. Binary classifications were derived for continuous variables using cut-off values determined by receiver operating characteristic (ROC) curves. Cut-off values were those values which discriminated between patients with normal or mildly reduced RV function and those with reduced RV function, and between patients with and without at least mild or greater TR/PR with the greatest sensitivity and specificity [30]. These binary classifications were then used in logistic regression. Statistical analysis was performed using SPSS 15.0 software (SPSS Inc., Chicago, IL). P-values are two-sided and considered significant when < 0.05.

## Results

### Patient characteristics

Median age of the patients was 63 years (range 33-83), 48% were men. Sixty patients (87%) had liver metastases. Fifty-nine patients (86%) had the carcinoid syndrome defined as the presence of flushing and/or diarrhea.

### RV valvular pathology

Forty patients had at least mild or greater TR/PR. Of these, 24 had mild, 9 had mild-to-moderate, and 7 had moderate or severe TR/PR. Only one patient in the entire group had pulmonary stenosis in addition to TR. None of the patients had tricuspid stenosis. Patients with at least mild or greater TR/PR were significantly older, had longer duration of illness, had a higher frequency of the carcinoid syndrome and had more numerous liver metastases than patients with no or minimal TR/PR (Table 1). Of the 40 patients with at least mild or greater TR/PR, 15 fulfilled the criteria for CHD. Of these, 14 had TR while one patient had only PR. One patient with moderate TR did not have any morphologic changes of the tricuspid valve and thus, was not included in the CHD group. Of note, U-5HIAA (20 [6-47] vs. 6 [2-25] μmol/mmol creatinine, p = 0.03) and CgA (26 [15-153] vs 16 [6-38] nmol/L, p = 0.02) levels were significantly elevated in the patients fulfilling the criteria for CHD which is in agreement with previous studies [2,4].

### RV function

Data on RV lateral systolic myocardial strain were available in all 69 patients with a mean value of -21% ± 5. Thirty-three patients had reduced RV function (strain > -20%, mean -16% ± 3), while 36 patients had normal or mildly reduced RV function (strain ≤ -20%, mean -25% ± 3). Of the 33 patients with reduced RV function, 8 (24%) had no or minimal, 21 (64%) had mild or mild-to-moderate, and 4 (12%) had moderate or severe TR/PR. RV strain values were significantly lower in the group with at least mild or greater TR/PR compared to those with no or minimal TR/PR (-20% ± 5 vs. -22% ± 5, p = 0.03). In addition, there was a significant association between reduced RV function and the degree of valvular regurgitation (p = 0.02) (Table 2). As shown in table 1, the patients with reduced RV function had significantly higher CgA levels and more numerous liver metastases and tended to have higher U-5HIAA levels than those with normal or mildly reduced RV function.

**Table 2:** Association Between Strain and Degree of Valvular Regurgitation*

	No/minimal TR/PR†	Mild/mild-to-moderat TR/PR	Moderate to severe TR/PR	Total Patients
**Normal/mildly reduced** **RV function‡**	21	12	3	36
**Reduced RV function**	8	21	4	33

**Total patients**	29	33	7	69

*Fisher's exact test, p = 0.02. †Tricuspid and/or pulmonary regurgitation. ‡Right ventricular function.

### CCN2 in relation to RV dysfunction in NET patients

**Figure 2:**
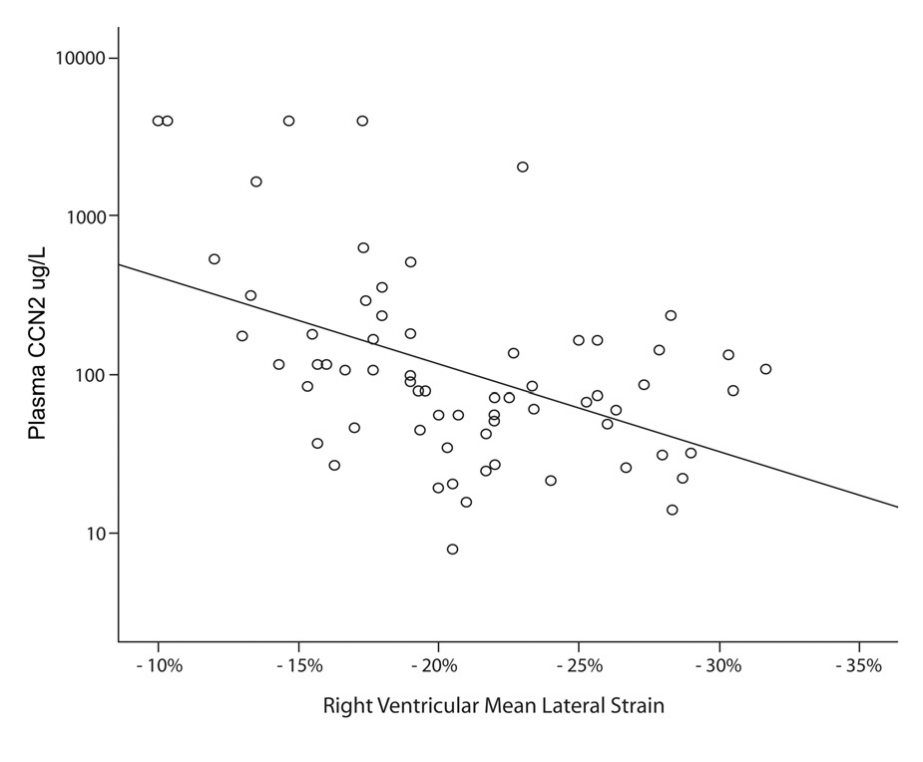
**Linear relationship between plasma connective tissue growth factor and right ventricular strain**. Scatter plot showing the inverse relationship between plasma connective tissue growth factor (CCN2) levels and right ventricular strain (linear regression, r = 0.47, p < 0.001). Due to the presence of extreme outliers, plasma CCN2 levels are plotted on the y-axis using a logarithmic (log10) scale.

**Figure 3:**
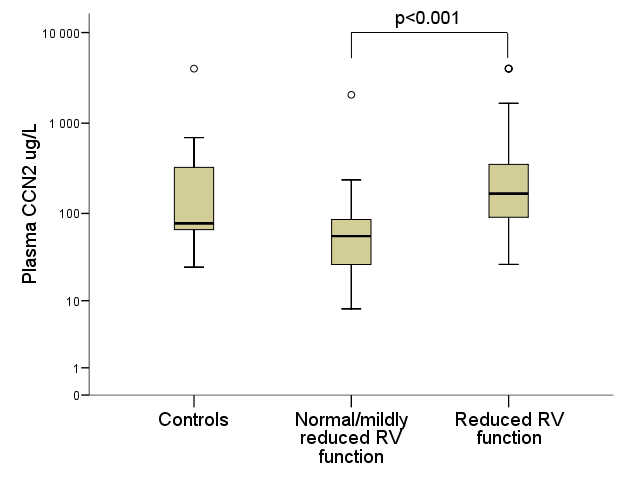
**Relationship between plasma connective tissue growth factor and right ventricular function**. Box plot demonstrating the relationship between plasma connective tissue growth factor (CCN2) levels and right ventricular (RV) function. Median, interquartile ranges, and maximum and minimum values are shown. Due to the presence of extreme outliers, plasma CCN2 levels are plotted on the y-axis using a logarithmic (log10) scale. Plasma CCN2 levels (μg/L) are significantly elevated in the patients with reduced RV function (strain > -20%), (Mann-Whitney U test, p < 0.001).

**Figure 4:**
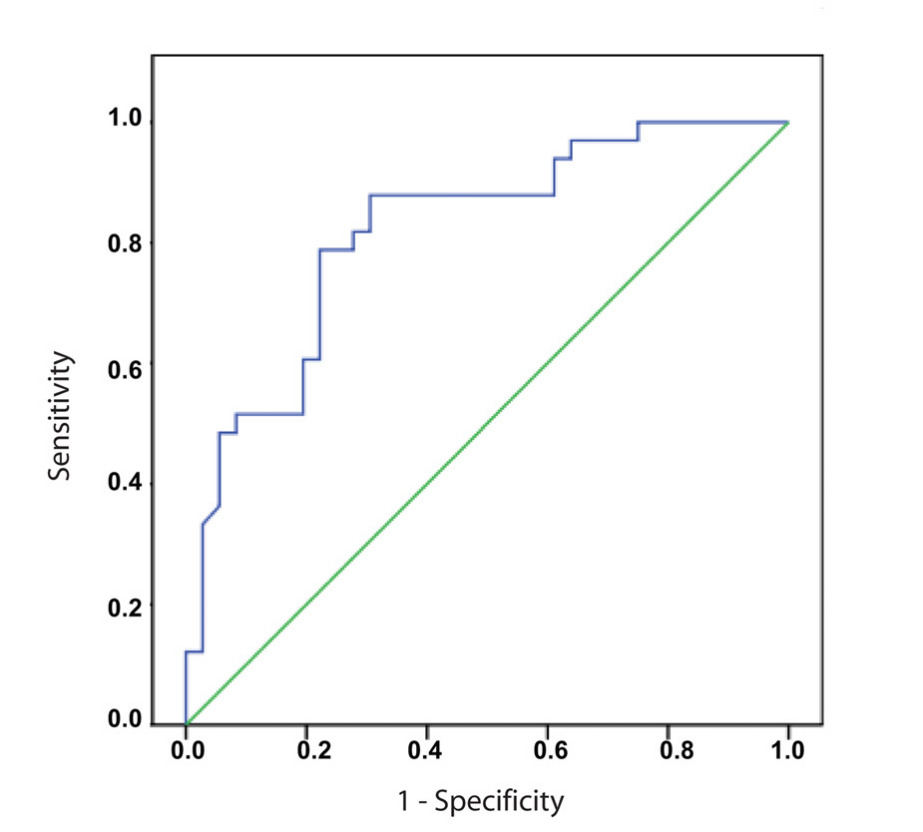
**Utility of plasma connective tissue growth factor as a predictor of reduced right ventricular function**. Receiver operating characteristic (ROC) curve depicting the ability of plasma connective tissue growth factor (CCN2) levels to predict the presence of reduced right ventricular (RV) function (AUC 0.82 [95% CI 0.72;0.92], p < 0.001). Plasma CCN2 levels ≥ 77 μg/L had 88% sensitivity and 69% specificity for detecting patients with reduced RV function.

No difference in the plasma levels of CCN2 was seen between the total patient group and sex- and age-matched healthy controls (87 [45-171] vs. 78 μg/L [59-347]), p = 0.59). There was also no significant difference in plasma CCN2 levels in relation to the presence (n = 15) or absence (n = 54) of CHD using current criteria based on valvular pathology (116 [74-180] vs. 82 μg/L [41-169], p = 0.20). However, several significant findings were revealed when analyzing the relationship between CCN2 and RV dysfunction by means of RV strain examination. First, in the patient group as a whole, there was a significant inverse correlation between RV function and plasma CCN2 levels (r = 0.47, p < 0.001) (Figure 2). In line with this, plasma CCN2 levels were significantly elevated in patients with reduced RV function compared to those with normal or mildly reduced RV function (p < 0.001) (Figure 3). As determined by ROC curve analysis, plasma CCN2 levels ≥ 77 μg/L had 88% sensitivity and 69% specificity for detecting patients with reduced RV function (AUC 0.82 [95% CI 0.72;0.92], p < 0.001) (Figure 4). Using univariate logistic regression, plasma CCN2 levels ≥ 77 μg/L (p < 0.001), CgA levels ≥ 22 nmol/L (p = 0.007) and number of liver metastases ≥ 8 (p = 0.01) were significantly associated with reduced RV function. In multivariate analysis, only CCN2 ≥ 77 μg/L (p < 0.001) and CgA ≥ 22 nmol/L (p = 0.04) remained independent predictors of reduced RV function (Table 3). Similar results were obtained using both forward and backward conditional stepwise methods.

**Table 3:** Independent Predictors of Reduced Right Ventricular Function*

	Univariate Analysis†	P-value	Multivariate Analysis	P-value
**CCN2 ≥ 77 μg/L‡**	16.48 [4.66;58.28]	**<0.001**	15.36 [4.15;56.86]	**<0.001**
**CgA ≥ 22 nmol/L§**	4.07 [1.46;11.32]	**0.007**	3.57 [1.03;12.29]	**0.04**
**No. mets ≥ 8**||	3.61 [1.33;9.83]	**0.01**		

*Logistic regression. †Results given as odds ratio (OR) with 95% confidence interval. ‡Connective tissue growth factor. §Chromogranin A (normal < 4 nmol/L). ||Number of liver metastases.

### CCN2 in relation to right heart valvular involvement in NET patients

**Figure 5:**
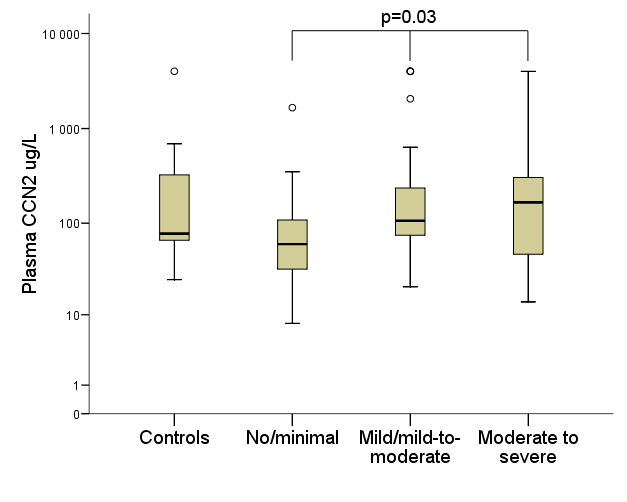
**Relationship between plasma connective tissue growth factor and degree of valvular regurgitation**. Box plot displaying the relationship between plasma connective tissue growth factor (CCN2) levels and the degree of tricuspid and/or pulmonary regurgitation. Median, interquartile range, and maximum and minimum values are shown. Due to the presence of extreme outliers, plasma CCN2 levels are plotted on the y-axis using a logarithmic (log10) scale. The highest plasma CCN2 levels were seen in the patients with moderate to severe right-sided valvular regurgitation (Kruskal-Wallis test, p = 0.03).

Plasma CCN2 levels for each of the categories of valvular involvement are given in table 4. Patients with at least mild or greater TR/PR had significantly higher plasma CCN2 levels compared to patients with no or minimal TR/PR (111 [69-279] vs 60 μg/L [32-112], p = 0.008). Furthermore, when the patients were divided into three groups according to the degree of TR/PR, there was a gradual rise in CCN2 levels, with the highest levels seen in those with moderate to severe TR/PR (p = 0.03) (Figure 5). Using cut-off values determined by ROC curve analysis, plasma CCN2 ≥ 86 μg/L, age ≥ 67 years, presence of ≥ 9 liver metastases and duration of illness ≥ 46 months were significantly associated with at least mild or greater TR/PR, as was presence of the carcinoid syndrome. In multivariate analysis, only plasma CCN2 ≥ 86 μg/L (p = 0.008), age ≥ 67 years (p = 0.001) and presence of ≥ 9 liver metastases (p = 0.04) remained independent predictors of the presence of at least mild or greater TR/PR (Table 5).

**Table 4:** Plasma CCN2 and Degree of Valvular Involvement

Degree of TR/PR*	# Patients	Plasma CCN2*ug/L
**Controls**	18	78 (59-347)
**None/minimal**	29	60 (32-112)
**Mild**	24	111 (57-309)
**Mild-to-moderate**	9	91 (71-165)
**Moderate**	4	130 (40-433)
**Severe**	3	166 (14-4000)

*Tricuspid and/or pulmonary regurgitation. *Connective tissue growth factor. Results given as median (interquartile range).

**Table 5:** Independent Predictors of Mild to Severe Tricuspid or Pulmonary Regurgitation*

	Univariate Analysis†	P-value	Multivariate Analysis	P-value
**CCN2 ≥ 86 μg/L‡**	5.45 [1.91;15.57]	**0.002**	7.76 [1.71;35.28]	**0.008**
**Age ≥ 67 years**	7.20 [2.27;22.80]	**0.001**	15.48 [3.00;79.89]	**0.001**
**No. mets ≥ 9§**	3.55 [1.27;9.23]	**0.02**	4.56 [1.06;19.63]	**0.04**
**Duration ≥ 46 mos||**	3.70 [1.34;10.21]	**0.01**		
**Carcinoid syndrome¶**	7.24 [1.41, 37.26]	**0.02**		

*Logistic regression. †Results given as odds ratio (OR) with 95% confidence interval. ‡Connective tissue growth factor. §Number of liver metastases. **||**Duration of illness in months. ¶Defined as flushing and/or diarrhea.

### Immunostaining of neuroendocrine tumors

**Figure 6:**
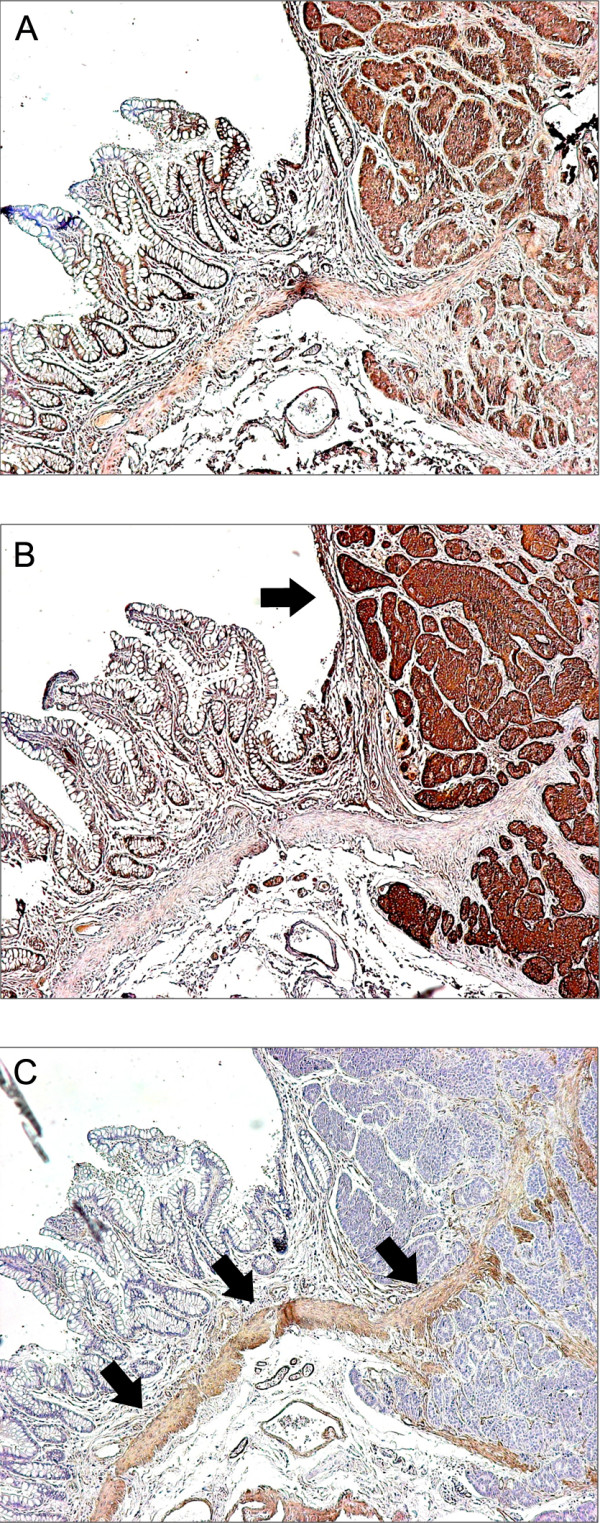
**Immunostaining of neuroendocrine tumors**. Representative photomicrographs demonstrating anti-connective tissue growth factor (CCN2) (panel A) immunostaining of gut carcinoid tumor tissue. CCN2 immunoreactivity was seen in cells corresponding to chromogranin A-positive tumor cells and gut smooth muscle (denoted by arrows in panels B and C, respectively). All sections were counterstained with hematoxylin. Magnification ×50.

Fairly strong CCN2 immunoreactivity was found in CgA positive (tumor) tissue obtained from both the gut and from the liver. Weaker immunostaining for CCN2 was seen in gut smooth muscle cells (Figure 6). Positive immunostaining was present in all seven biopsies examined, that is from both patients with and without reduced RV function and from patients with and without at least mild or greater TR/PR.

## Discussion

**Figure 7:**
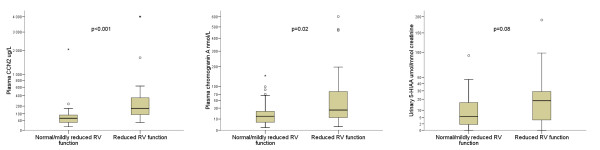
**Relationship between plasma connective tissue growth factor, chromogranin A and urinary 5-hydroxyindoleacetic acid and right ventricular function**. Box plots displaying the relationship between plasma connective tissue growth factor (CCN2), chromogranin A (CgA) and urinary 5-hydroxyindoleacetic acid (U-5HIAA) levels and right ventricular (RV) function. Median, interquartile ranges, and maximum and minimum values are shown. Due to the presence of extreme outliers, values are plotted on the y-axis using a logarithmic (log e) scale. P-values are determined using Mann-Whitney U test.

In the present study, a significant inverse correlation was found between RV function and plasma CCN2 levels. Furthermore, patients with reduced function of the right lateral ventricular wall had elevated plasma CCN2 levels even in the absence of valvular regurgitation. When the patients were grouped according to the presence or absence of valvular regurgitation, plasma CCN2 levels were significantly elevated in patients with at least mild or greater TR/PR compared to those patients with no or minimal TR/PR. Importantly, an elevated plasma CCN2 level was an independent predictor of both reduced RV function and the presence of any right-sided valvular regurgitation. Moreover, patients with more advanced stages of frank CHD, that is moderate or severe valvular involvement with associated morphologic changes, had the highest CCN2 levels. Thus, although we found no association between CCN2 and the presence of CHD based on current criteria, our findings suggest that plasma levels of CCN2 could potentially be a marker of early cardiac fibrosis in patients with NETs reflecting both mural and valvular pathology. As shown in figure 7, CCN2 compares favorably to both CgA and U-5HIAA with regard to predicting the presence of reduced RV function.

The CCN2 protein is thought to consist of four separate modules. Module 1 is identical to insulin-like growth factor binding protein, module 2 consists of a chordin-like cysteine-rich domain, module 3 is composed of a thrombospondin type 1 domain and module 4 is designated as the CT module or cysteine knot. Modules 1 and 2 make up the N-terminal domain and are linked by a hinge region to the C-terminal domain which is made up of modules 3 and 4 [31,32]. The ELISA analysis used in our study detects C-terminal CCN2 which translates into detection of both C-terminal fragments and whole CCN2. Our analysis is not able to detect N-terminal CCN2 fragments. Other studies have evaluated the utility of N-terminal CCN2 as a marker of intraocular fibrosis and in scleroderma. In these studies, neither C-terminal nor whole CCN2 were elevated in disease. As as result, it has been postulated that during a fibrotic response, *in vivo *CCN2 is cleaved resulting in N-terminal fragments which are excreted into the extracellular space. The C-terminal fragment may remain attached to the cell membrane or be internalized and degraded after exerting its effects. These results suggest that circulating N-terminal CCN2 may be a better marker of fibrosis [31,33]. Other studies, however, have shown significant elevations in whole CCN2 in patients compared to controls [34]. It is possible that the measurement of different CCN2 fragments may reveal varying strengths regarding the ability to predict the presence of disease. Nonetheless, we have demonstrated a significant difference in plasma CCN2 levels (C-terminal and/or whole) in patients with and without reduced RV function and in patients with and without any valvular regurgitation. Based on the above studies, it is conceivable that the difference would have been even more significant had we measured the N-terminal fragment, representing a possible limitation of our study.

It is of interest to note that in our patients, decreased RV function was not associated with age while valvular regurgitation was. As both decreased RV function and valvular changes may be involved in the fibrotic process leading to CHD, it is unclear why we found this discrepancy. It is possible that valvular structures are more predisposed to degenerative changes due to natural aging than myocardial tissue. Thus, the combination of profibrotic conditions and increased age in patients with neuroendocrine tumors may make such patients especially at risk for valvular dysfunction.

CCN2 is an immediate early gene product and some data suggest that CCN2 expression rapidly increases in the early stages of fibrotic processes, and then continues to be elevated throughout the chronic phase of fibrosis [12,13]. This may potentially explain our findings of elevated CCN2 levels even in patients with decreased RV function in the absence of right-sided valvular changes, in other words in patients with possibly the earliest stages of CHD. In contrast to earlier findings on serum levels of CCN2 in NET patients [16], we did not show any difference in plasma CCN2 levels between the total patient group and controls. However, as platelets contribute significantly to circulating CCN2 levels through platelet activation [34-36], it may be preferential to measure this protein in platelet-poor plasma to avoid the influence from platelet-mediated release of CCN2 during *ex vivo *coagulation of serum. Thus, our results may not be comparable to studies assessing serum CCN2 levels.

As shown in figure 3, there appears to be a trend towards lower plasma CCN2 levels in the patients with normal/mildly reduced RV function compared to controls although this difference is not significant (p = 0.054, Kruskal-Wallis with post-hoc analysis). This suggests that the control group has higher CCN2 values than would be expected in a group of healthy individuals. There are several possible reasons for this. One is that the number of individuals in the control group is small likely making an appropriate comparison difficult. Secondly, several of the controls had very high levels, including one over 4000 ug/L. In a group of only 18 controls, outliers have a greater effect on the median and interquartile range than in a larger group. In agreement with our findings, a recent study evaluating the role of CCN2 as a marker of fibrogenesis in liver disease also demonstrated extreme outliers in the control group. The control group, however, was much larger (n = 74) and they were able to show a significant difference between controls and patients [34]. The small size of the control group is a recognized limitation of our study. Finally, although the control individuals were healthy based on self-reporting, the possibility also exists that some of them may have had undiagnosed occult diseases which affect CCN2 levels. Nonetheless, despite these limitations in the selection of the control group, within the patient group it is clear that there is a significant difference between plasma CCN2 levels in patients with reduced RV function compared to those with normal/mildly reduced RV function. Moreover, the gradual rise along with increasing mural and valvular pathology, clearly suggest a link between CCN2 and RV dysfunction in NETs.

The cellular source of raised CCN2 levels in our group of patients is currently not known. One possibility is that the tumor cells themselves express and secrete CCN2 into the circulation which then exerts its effects upon a distant organ such as the heart. We showed positive immunostaining for CCN2 in tissue from both primary small intestinal tumors and liver metastases which is in agreement with previous findings [16], but we could not demonstrate any gross difference in the staining of tumor tissue in relation to strain or valvular pathology. However, immunohistochemistry was performed in relatively few patients, and future studies should also use more quantitative methods for assessment of CCN2 in tissue samples.

Another possibility is that CCN2 expression is induced locally in the heart by some other circulating factor, with one likely candidate being serotonin (5-hydroxytryptamine, 5-HT). NETs, especially those arising from the small intestine, secrete vasoactive substances such as serotonin into the circulation. It is thought that exposure of the right heart to these vasoactive substances results in the characteristic fibrotic lesions seen in CHD. It has been shown that serotonin directly, via the Rho signaling pathway [37], and indirectly, via induction of transforming growth factor (TGF)-β [38], induces CCN2 expression in renal mesangial cells via the Gq-coupled 5-HT_2A _receptor. It is not known whether serotonin directly induces CCN2 expression in the heart. It is known, however, that serotonin receptors (subtypes 5-HT_1B, 1D, 2A, 2B_) are normally expressed in human heart valve interstitial cells [39,40], and it has been shown that 5-HT_2B _receptors are expressed in human cardiac fibroblasts and cardiomyocytes [41]. Moreover, in CHD lesions, 5-HT_1B _receptors [42] as well as TGF-β1 and TGF-β3 are upregulated [43]. Also of interest to our study, CCN2 expression in normal rat hearts has been shown to be mainly expressed in endothelial cells and fibroblasts and that TGF-β induces CCN2 mRNA expression in rat cardiac fibroblasts [12]. Although we do not have mechanistic data, it is tempting to hypothesize that circulating serotonin may induce CCN2 expression in cardiac fibroblasts directly as well as via induction of TGF-β leading to increased collagen deposition and fibrosis in both mural and valvular endocardium. Whether or not elevated levels of CCN2 in neuroendocrine patients with decreased RV function (possibly reflecting fibrosis in the mural endocardium) and/or valvular involvement (fibrosis in the valvular endocardium) is the result of circulating CCN2 acting directly on cardiac fibroblasts or, more probably, is the result of local increased production due to stimulation by serotonin, remains to be seen. Nonetheless, based on the potent fibrogenic properties of CCN2 as well as its relationship to cardiac fibrosis, we suggest that CCN2 is not only a marker but may also be a mediator in the development of mural and valvular pathology in patients with NETs.

Five of the patients in our cohort had extremely elevated plasma CCN2 levels (> 4000 μg/L), a finding also documented in other studies [34]. It has been demonstrated that polymorphisms in the CCN2 promoter exist in normal individuals and that increased prevalence of certain polymorphisms may be associated with fibrotic diseases such as systemic sclerosis [44]. However, it is not known if certain polymorphisms are associated with higher plasma CCN2 levels. Future studies should examine whether or not any relationship between certain CCN2 polymorphisms and the development of RV pathology in NET patients can be demonstrated.

It is well-known that mild right-sided valvular regurgitation can be a normal physiological variant in healthy persons. Thus, we cannot say the presence of mild TR/PR in our patients necessarily represents a pathological process. However, we hypothesize that our patients with mild TR/PR and elevated CCN2 levels, as well as those patients with decreased RV function (even in the absence of valvular changes) and elevated CCN2 levels, are in the process of developing frank CHD, but future studies are needed to confirm this hypothesis.

## Conclusion

Elevated plasma CCN2 levels are associated with RV dysfunction in patients with NETs, including those not fulfilling the criteria for CHD. Elevated CCN2 levels are also associated with the presence of at least mild or greater TR/PR. Our data indicate a role for CCN2 in the development and progression of cardiac fibrosis related to NETs, and suggest that plasma CCN2 may serve as a marker for the earliest stages of CHD.

## Abbreviations

**CgA**: chromogranin A; **CHD**: carcinoid heart disease; **CCN**: Cyr61/CEF-10, CTGF/Fisp-12 and Nov family of matricellular proteins; **CCN2**: connective tissue growth factor; **NET**: neuroendocrine tumor; **ROC curve**: receiver operating characteristic curve; **RV**: right ventricle; **TGF-β**: transforming growth factor-beta; **TR/PR**: tricuspid and/or pulmonary regurgitation; **U-5HIAA**: urinary 5-hydroxyindoleacetic acid; **5-HT**: 5-hydroxytryptamine.

## Competing interests

The authors declare that they have no competing interests.

## Authors' contributions

DSB was involved in conception and design of the study, was responsible for patient inclusion, acquisition of material and clinical data, performed statistical analysis, and drafted the manuscript including preparation of figures and tables. JG and HA were responsible for the enzyme-linked immunosorbent assay and were involved in interpretation of data. KHH and LGS were involved in design of the study, performed strain analysis of the echocardiographic studies and interpreted data. SA and TE were involved in conception and design of the study, performed echocardiographic studies and interpreted data. ETE was involved in conception and design of the study, inclusion of patients and interpretation of data. EØ was responsible for the immunohistochemical studies and was involved in interpretation of data. PA was involved in conception and design of the study and interpretation of data. All authors read and were involved in critical revision of the article. All authors approved the final manuscript.

## Pre-publication history

The pre-publication history for this paper can be accessed here:

http://www.biomedcentral.com/1471-2407/10/6/prepub
